# The Correlations of Plasma and Cerebrospinal Fluid Amyloid-Beta Levels with Platelet Count in Patients with Alzheimer's Disease

**DOI:** 10.1155/2018/7302045

**Published:** 2018-10-15

**Authors:** Hao-Lun Sun, Wei-Wei Li, Chi Zhu, Wang-Sheng Jin, Yu-Hui Liu, Fan Zeng, Yan-Jiang Wang, Xian-Le Bu

**Affiliations:** Department of Neurology and Centre for Clinical Neuroscience, Daping Hospital, Third Military Medical University, Chongqing, China

## Abstract

**Purpose:**

Recent study shows that blood-derived amyloid-beta (A*β*) can induce cerebral amyloidosis and is involved in the pathogenesis of Alzheimer's disease (AD). The vast majority of blood A*β* is generated from platelet. Whether blood A*β* levels are associated with the count of platelets remains unknown.

**Methods:**

58 clinically diagnosed AD patients, 18 ^11^C-PIB-PET diagnosed AD patients, and 61 age- and gender-matched cognitively normal controls were included to analyze the correlation of plasma A*β* levels with platelet count. 13 AD patients and 40 controls with cerebrospinal fluid (CSF) samples were included to further analyze the correlation of CSF A*β* levels with platelet count. A*β*40 and A*β*42 levels in plasma and CSF were measured by ELISA kits.

**Results:**

The plasma A*β*42 level was positively correlated with platelet count in both AD patients and control group, especially in AD patients with positive PIB-PET, while there was no correlation as to A*β*40. The CSF A*β* levels also had no significant correlation with platelet count.

**Conclusion:**

It suggests that platelets may be involved in the pathogenesis of AD and become a potential peripheral biomarker for AD.

## 1. Introduction

Alzheimer's disease (AD) is the most common neurodegenerative disorder in the elderly. It causes cognitive deficits and memory dysfunction that usually starts slowly and worsens over time. Amyloid-beta (A*β*) has been proven to play a crucial role in pathogenesis of AD. Production and aggregation of the A*β* peptide give rise to the pathology of AD [1], and A*β* deposition in the brain parenchyma causes neuritic plaques, one of the neuropathological hallmarks of AD. Recent study has shown that blood A*β* could enter brain and lead to the occurrence of AD, suggesting that AD may be a systemic disease [2]. Platelets express amyloid precursor protein (APP), *β*-secretase, and *γ*-secretase, and thus it can generate A*β* [3, 4]. It has been shown that almost 90% of the blood A*β* originated from platelet [5]. Further, the activity of platelet membrane *β*-secretase in AD patients is significantly increased [6, 7]. Platelet activation may lead to the deposition of A*β*40 in the wall of brain vessels, which results in cerebral amyloid angiopathy (CAA) [8]. Thus, platelets are closely related to the pathogenesis of AD and may be a potential biomarker for early diagnosis of AD [9, 10]. However, there is a lack of clinical research on the relationship between platelets and A*β*. In the present study, we aim to investigate whether A*β* levels are correlated with the platelet count.

## 2. Methods

### 2.1. Study Population

AD patients were recruited from Chongqing Daping Hospital from May to December in 2017. Age- and gender-matched controls with normal cognition were randomly recruited from the hospitals during the same time. Subjects were excluded for the following reasons: (1) a family history of dementia; (2) a concomitant neurologic disorder that could potentially affect cognitive function or other types of dementia; (3) severe cardiac, pulmonary, hepatic, or renal diseases or any type of tumor; (4) enduring mental illness (e.g., schizophrenia); (5) hematological diseases or other diseases that could affect the number of platelets (e.g., essential thrombopenia); (6) use antiplatelet drugs recently (e.g., Aspirin). For the subjects with hypertension, their systolic blood pressure was controlled below 140 mmHg and the diastolic blood pressure was controlled below 90 mmHg by antihypertensive medication, such as captopril, valsartan, and nifedipine. As to diabetes, the fasting blood glucose was controlled between 5 and 7 mmol/L, and the postprandial blood glucose was controlled between 8 and 11 mmol/L by hypoglycemic agent (e.g., metformin, acarbose, and insulin). For the participants with hyperlipidemia, the blood low density lipoprotein ranges between 3.45 and 4.42 mmol/L, and some were given statins after evaluating against the balancing of risks and benefits of the medications. The study was conducted in accordance with the Declaration of Helsinki and International Conference on Harmonisation Guidelines for Good Clinical Practice, and it was approved by Institutional Review Board of Daping Hospital.

### 2.2. AD Diagnosis and Sampling

The clinical assessment and diagnosis of AD dementia were performed following the protocol we used before [11]. In brief, the demographic data and medical history (such as hypertension, coronary heart disease, and diabetes mellitus) were collected. The cognitive and functional status were assessed based on a neuropsychological battery, and diagnosis of AD was made according to the criteria of National Institute of Neurological and Communicative Diseases and Stroke/AD and Related Disorders Association following the protocols we used before [11]. In addition, 18 people were administered with A*β* positron emission tomography (PET) inspection with Pittsburg compound B to detect and quantify A*β* deposition in the brain. Fasting blood was collected between 06:00 and 07:00 to avoid the potential circadian rhythm influence. The blood samples were centrifuged at once after sampled and then stored at −80°C until use. Some participants underwent lumbar puncture (CON, n=40; AD, n=13) to obtain cerebrospinal fluid (CSF). The CSF samples were centrifuged at 2000g at 4°C for 10 min, and the aliquots were then immediately frozen and stored at − 80°C until use. The informed consent was obtained before the acquisition of the blood and CSF samples.

### 2.3. Measurements of Platelet and A*β* Levels

The count of platelet was measured using standard laboratory methods in the Clinical Laboratory, Daping Hospital, Chongqing, China. The human A*β* enzyme-linked immunosorbent assay (ELISA) kits (Invitrogen) were used to measure A*β*40 and A*β*42 levels in plasma and CSF. All of the ELISA measurements were performed in accordance with the manufacturers' instructions. All the samples and standards had received reduplicated measurements and statistical analyses.

### 2.4. Statistical Analysis

The differences in demographic characteristics, platelet count, and A*β* levels between the groups were assessed with two-tailed independent t-tests, Mann-Whitney U test, or Chi-square test. Spearman correlation analyses were used to examine the correlations between platelet count and the A*β* levels. The data are expressed as the mean± standard deviation (SD). All hypothesis testing was two-sided, and p< 0.05 was defined as statistically significant. The computations were performed with SPSS version 20.0 (SPSS Inc., Chicago, IL, USA)

## 3. Results

### 3.1. Characteristics of the Study Population

As shown in Table 1, the study consisted of 58 clinically diagnosed AD patients, 18 ^11^C-PIB-PET diagnosed AD patients and 61 age- and gender-matched cognitively normal controls. AD patients and controls were similar in educational level (*p1*=0.225,* p2*=0.550), and there were no significant differences in the comorbidity of hypertension, diabetes mellitus, cardiovascular disease, and hyperlipidemia. No significant difference was found in the count of platelet between AD patients and controls (*p1*=0.478). AD patients with positive PIB-PET had slightly elevated platelets, but it was still not statistically significant (*p2*=0.275). The MMSE score of AD patients was lower than that of the controls (p1<0.001, p2<0.001). The patients with AD were generally higher in CDR (*p1*<0.001,* p2*<0.001).

### 3.2. Correlations of Plasma A*β* Levels with Platelet Count in AD Patients, Normal Controls, and All Cases

**Figure 1:**
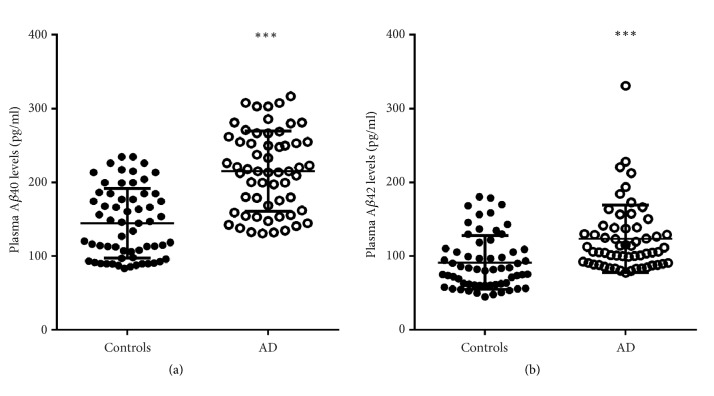
**Comparison of the plasma A** **β** **levels between the controls and patients with AD.** *∗∗∗* denotes p< 0.001.

**Figure 2:**
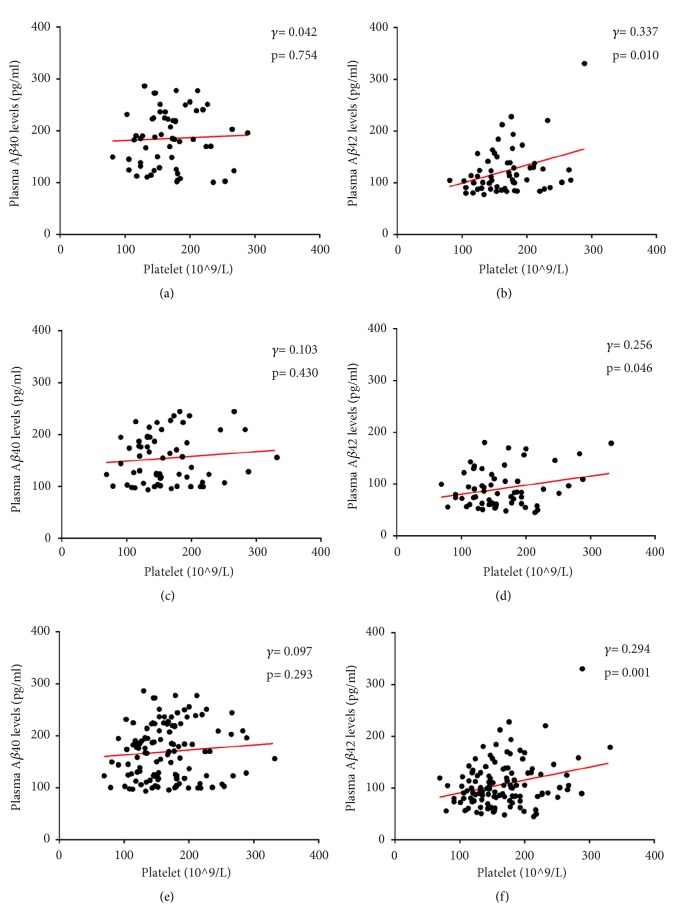
Correlations of plasma A*β* levels with platelet count in AD patients ((a) and (b)), normal controls ((c) and (d)), and all cases ((e) and (f)).

The AD patients had significant higher levels of both plasma A*β*40 (215.25±54.26 pg/ml versus 144.62±47.20 pg/ml, p< 0.001) and A*β*42 (123.48±45.89 pg/ml versus 91.35±36.39 pg/ml, p< 0.001) than the control group (Figure 1). There was no correlation between plasma A*β*40 level and platelet count in AD patients (*γ*= 0.042, p= 0.754), the controls (*γ*= 0.103, p= 0.430), or all cases (*γ*= 0.097, p= 0.293) (Figures 2(a), 2(c), and 2(e)), while plasma A*β*42 level had significantly positive correlation with platelet count in AD patients (*γ*= 0.337, p= 0.010), controls (*γ*= 0.256, p= 0.046), and all cases (*γ*= 0.294, p= 0.001) (Figures 2(b), 2(d), and 2(f)).

### 3.3. Correlation of Plasma A*β*42 Levels with Platelet Count in AD Patients with Positive PiB-PET

**Figure 3:**
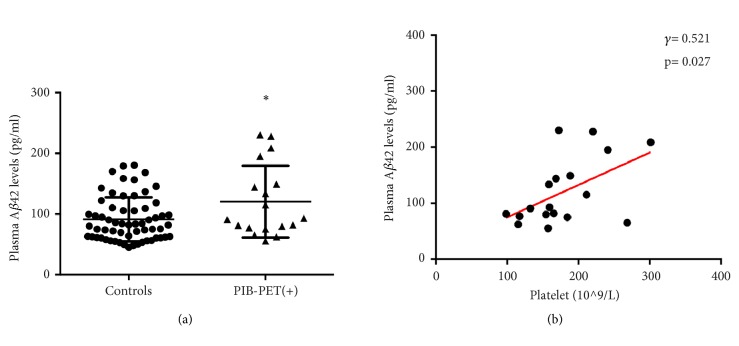
**Correlation of plasma A** **β** **42 levels with platelet count in AD patients with PiB-PET (+).** *∗* denotes p< 0.05.

In order to test the findings further, we analyzed the correlation in PiB-PET positive AD patients. Just as shown in Figure 3(a), the plasma A*β*42 level was significantly increased in PiB-PET positive AD patients, and it was also positively correlated with platelet count (*γ*= 0.521, p= 0.027) (Figure 3(b)). These further indicate that blood A*β*42 levels may raise with the increase of platelet count.

### 3.4. Correlations of CSF A*β* Levels with Platelet Count in AD Patients and Normal Controls

**Figure 4:**
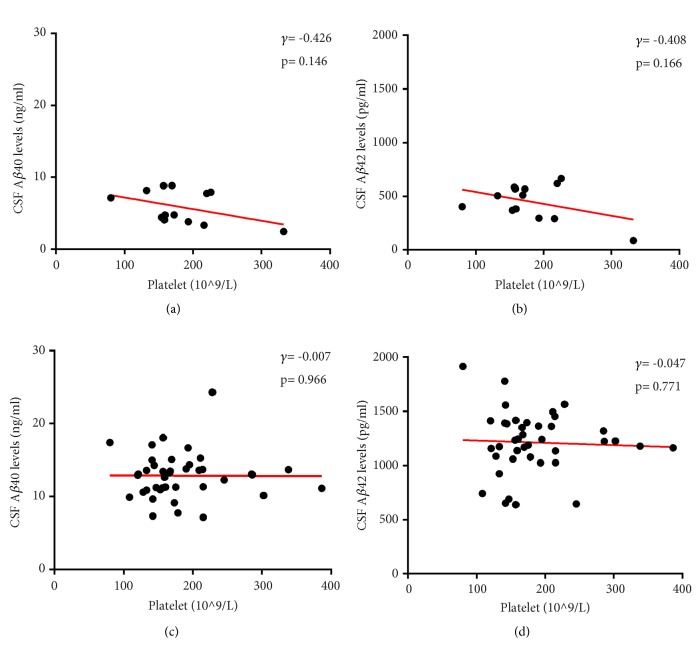
Correlations of CSF A*β* levels with platelet count in AD patients ((a) and (b)) and normal controls ((c) and (d)).

Then CSF were collected from 13 AD patients and 40 age- and gender-matched controls to further analyze the correlation of CSF A*β* levels with platelet count. As shown in Supplemental Table 1, there were no significant differences in the comorbidity of hypertension, diabetes mellitus, cardiovascular disease, and hyperlipidemia between two groups. Platelet counts also showed no difference. The CSF A*β*40 (5.88±2.29 ng/ml versus 12.87±3.18 ng/ml, p< 0.001) and A*β*42 (449.58±163.69 pg/ml versus 1212.17±285.09 pg/ml, p< 0.001) levels were significantly decreased in AD patients (Supplemental Figure 1). However, there were no correlations of CSF A*β*40 or A*β*42 levels with platelet count in either AD or control group (Figure 4).

## 4. Discussion

To our knowledge, this is the first research to investigate the association between plasma and CSF A*β* levels and platelet count. In this study, we found that the blood A*β*42 levels were positively correlated with platelet count, especially in AD patients with positive PiB-PET, while there was no significant correlation of blood A*β*40 or CSF A*β* levels with platelet count.

According to the previous point of view, brain A*β* is primarily produced by the transmembrane glycoprotein APP in neuron. Platelets, containing *β*- and *γ*-secretase, are the main peripheral source of APP protein and can generate A*β* in a mechanism similar to neurons [12, 13]. It is estimated that nearly 90% of blood A*β* is generated from platelets, which is mainly A*β*40 [5]. However, whether blood A*β* levels are associated with platelet count remains unknown. In this study, we further found a positive correlation between blood A*β*42 levels and platelet count, which is consistent with previous view platelet being the primary source of A*β*.

The lack of correlation between CSF A*β* levels and platelet count indicates that platelet count may not affect brain A*β* burden. The significant association between plasma A*β* levels and platelet count implies that platelet count may be not only involved in peripheral A*β* production but also in peripheral A*β* clearance. It has been shown that platelets regulate soluble A*β* into fibrillar structures by the absorption of apoptotic platelets [14] which leads to platelet activation and adhesion that also mediates the occurrence and development of cerebral amyloid angiopathy (CAA), which is common in AD. It is still not certain of why plasma A*β*42 but not A*β*40 was correlated with platelet count, but we think there are several possible reasons. One possible explanation is that A*β*40 is more readily degraded by plasma proteases such as insulin-degrading enzyme, neprilysin, angiotensin-converting enzyme, and metalloproteases that are abundant in the blood circulation [15]. A*β*42 is more resistant to degradation because it is more aggregating and adhesive. Additionally, increased platelet count may make A*β*42 more difficult to be cleared in the periphery [16]. Activated platelets also make A*β*42 resistant to be degraded [6]. Therefore, there is a positive correlation of plasma A*β*42 level with the number of platelets.

Previous studies also showed that the membrane *β*-secretase activity of platelet is increased by 24% in patients with mild cognitive impairment and by 17% in those with AD [6, 17, 18]. Therefore, the dysfunctions of APP metabolism might be a systemic problem in AD. There is no significant difference in the count of platelet between AD and normal control group. However, AD patients with positive PIB-PET tend to have increased platelet count. The increased activity of platelet *β*-secretase in AD patients could lead to the overproduction of A*β*, which is probably responsible for the higher blood A*β* levels in AD. It has been generally accepted that A*β* deposited in the brain originates from the brain tissue itself. However, our recent study proved that blood A*β* is capable of crossing the blood brain-barrier and inducing AD-type pathologies [2]. Since platelets contribute to the mainly blood A*β*. It is probably that the A*β* derived from platelet is contributory to AD pathogenesis.

This study implies that platelet may play a role in the pathogenesis of AD, which further indicates that AD is a systemic disease. Whether the disorders increasing platelet activity or platelet count could raise AD risk deserves further investigation. Notability, this is an observational study that we cannot determine the effect of platelets count and A*β* levels on AD progression. Longitudinal studies are needed to better clarify the impact of the dynamic changes of platelets and A*β* on AD in the future. In addition, we need to increase the number of AD patients with positive PiB-PET to better verify the difference in platelet count between AD and the controls.

In conclusion, our study showed that blood A*β*42 level was positively correlated with platelet count. It suggests that platelets may be involved in the pathogenesis of AD and become a potential biomarker for AD.

## Figures and Tables

**Table 1:** Characteristics of the study population.

	CON (n=61)	AD (n=58)	PIB-PET(+)-AD (n=18)	*P1 *value (AD vs. CON)	P2 value (PET vs. CON)
Age (years)	69.16±11.55	71.52±10.71	68.22±8.00	0.252	0.748
Female (%)	30 (49.18)	30 (51.72)	9 (50.00)	0.781	0.951
Education years	9.13±3.58	7.89±4.42	9.56±4.59	0.225	0.550
Hypertension (%)	14 (22.95)	11 (18.97)	3 (16.67)	0.594	0.807
Diabetes mellitus (%)	2 (3.28)	4 (6.90)	0	0.629	>0.999
Cardiovascular disease (%)	9 (14.75)	5 (8.62)	1 (5.56)	0.299	0.530
Hyperlipidemia (%)	5 (8.20)	4 (6.90)	0	>0.999	0.481
Platelet count	162.57±53.00	168.97±44.32	178.22±53.40	0.478	0.275
MMSE	27.17±2.75	16.48±6.51	12.17±5.76	<0.001	<0.001
CDR	0	1.86±0.86	2.08±0.88	<0.001	<0.001

Data given as mean ± SD unless otherwise stated. MMSE, Minimental State Examination; CDR, Clinical Dementia Rating. P value, two-tailed independent t-tests, Mann-Whitney U test, or Chi-square test as appropriate.

## Data Availability

The data used to support the findings of this study are available from the corresponding author upon reasonable request.

## Supplementary Materials

Supplementary Materials Supplemental Table 1 showed the characteristics of the participants with CSF samples. Supplemental Figure 1 showed the CSF A*β* levels of the controls and patients with AD.
