# Supplementary material for: The Correlations of Plasma and Cerebrospinal Fluid Amyloid-Beta Levels with Platelet Count in Patients with Alzheimer's Disease
Source: Biomed Res Int. 2018 Oct 15;2018:7302045. doi: 10.1155/2018/7302045 (PMC6205107; doi:10.1155/2018/7302045)

**Title:** The correlations of plasma and cerebrospinal fluid amyloid-beta levels with platelet count in patients with Alzheimer’s disease

Hao-Lun Sun ^1^, Wei-Wei Li ^1^, Chi Zhu ^1^, Wang-Sheng Jin ^1^, Yu-Hui Liu ^1^, Fan Zeng ^1^, Yan-Jiang Wang ^1^, Xian-Le Bu ^1,*^

^1^ Department of Neurology and Centre for Clinical Neuroscience, Daping Hospital, Third Military Medical University, Chongqing, China.

^*^ To whom correspondence should be addressed: Dr. Xian-Le Bu, email: buxianle@sina.cn, telephone: +86 23 68757850

**Running title:**Correlation of amyloid-beta and platelet

**Conflict of interest**
None

**Supplemental table 1. Characteristics of the participants with CSF samples**

|  | CON (n=40) | AD (n=13) | *P* value |
| --- | --- | --- | --- |
| Age（years） | 65.08±12.72 | 62.08±7.20 | 0.425 |
| Female (%) | 30 (75) | 7 (53.85) | 0.273 |
| Education years | 8.15±3.91 | 8.50±4.57 | 0.803 |
| Hypertension (%) | 7 (17.5) | 3 (23.08) | 0.970 |
| Diabetes mellitus (%) | 1 (2.5) | 0 | >0.999 |
| Cardiovascular disease (%) | 1 (2.5) | 1 (7.69) | 0.987 |
| Hyperlipidemia (%) | 0 | 1 (7.69) | 0.550 |
| Platelet count | 184.05±63.61 | 182.15±59.75 | 0.924 |
| MMSE | 28.53±2.01 | 12.15±4.63 | <0.001 |
| CDR | 0 | 2.08±0.86 | <0.001 |

Data given as Mean±SD unless otherwise stated. Abbreviations: MMSE, Mini-mental State Examination; CDR, Clinical Dementia Rating. P value, two-tailed independent t-tests, Mann-Whitney U test or Chi-square test as appropriate.

**Supplemental figure 1. Comparison of the CSF Aβ levels between the controls and patients with AD.** ***denotes p< 0.001.


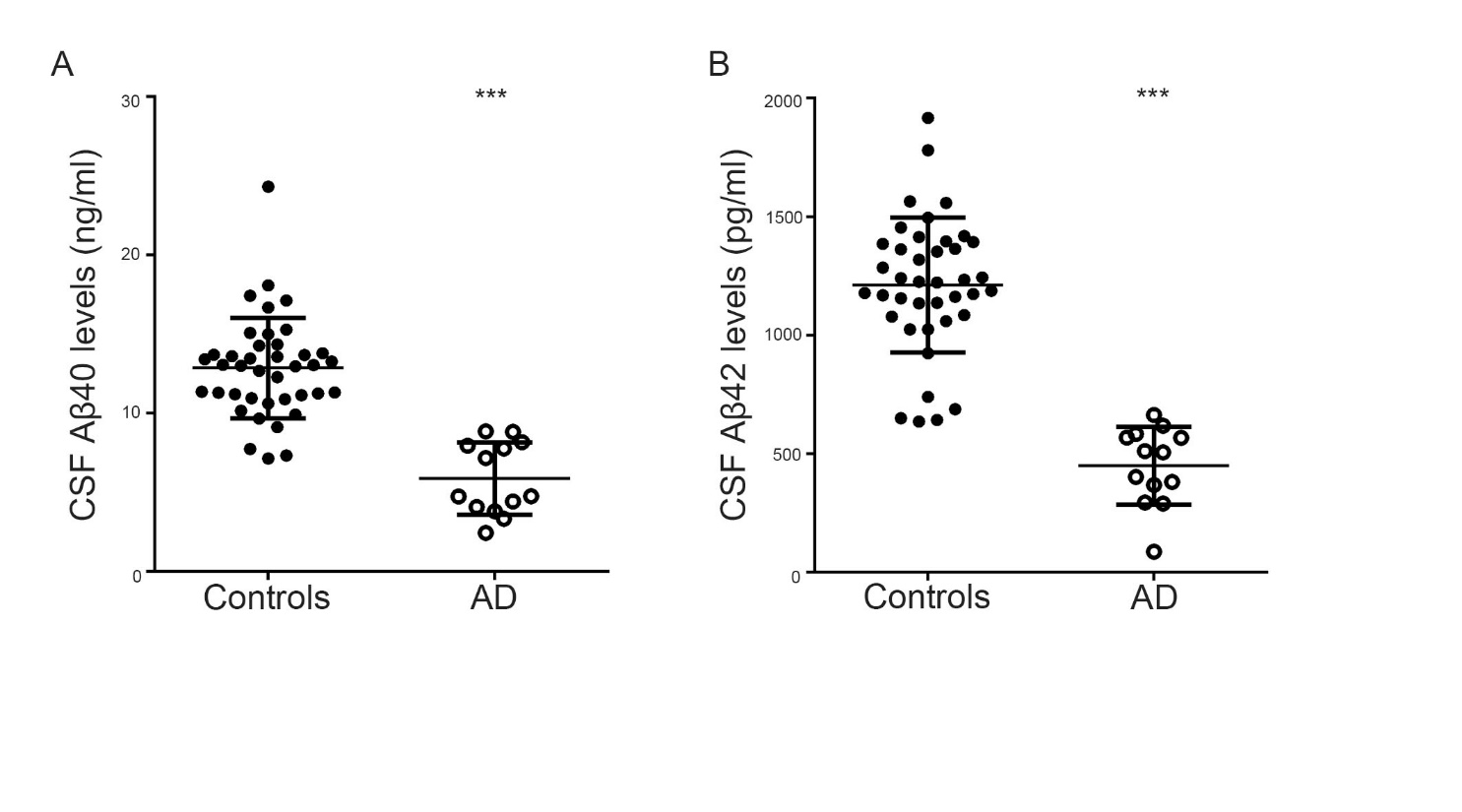

Supplement: Supplementary Materials — Supplemental Table 1 showed the characteristics of the participants with CSF samples. Supplemental Figure 1 showed the CSF Aβ levels of the controls and patients with AD. [file 7302045.f1.docx]
